# Numerical Study on Electrode Design for Rodent Deep Brain Stimulation With Implantations Cranial to Targeted Nuclei

**DOI:** 10.3389/fncom.2021.631188

**Published:** 2021-02-02

**Authors:** Konstantin Butenko, Rüdiger Köhling, Ursula van Rienen

**Affiliations:** ^1^Institute of General Electrical Engineering, University of Rostock, Rostock, Germany; ^2^Oscar-Langendorff-Institute of Physiology, Rostock University Medical Center, Rostock, Germany; ^3^Interdisciplinary Faculty, University of Rostock, Rostock, Germany; ^4^Department Life, Light & Matter, University of Rostock, Rostock, Germany

**Keywords:** deep brain stimulation, electric field modeling, electrode design, neural activation, rodent model

## Abstract

The globus pallidus internus and the subthalamic nucleus are common targets for deep brain stimulation to alleviate symptoms of Parkinson's disease and dystonia. In the rodent models, however, their direct targeting is hindered by the relatively large dimensions of applied electrodes. To reduce the neurological damage, the electrodes are usually implanted cranial to the nuclei, thus exposing the non-targeted brain regions to large electric fields and, in turn, possible undesired stimulation effects. In this numerical study, we analyze the spread of the fields for the conventional electrodes and several modifications. As a result, we present a relatively simple electrode design that allows an efficient focalization of the stimulating field in the inferiorly located nuclei.

## Introduction

The exact mechanisms of deep brain stimulation (DBS) for Parkinson's disease and dystonia treatment still remain unclear. To explore these mechanisms, often experimental approaches involving stimulation in rodent models such as 6-hydroxydopamine treated rats and genetically dystonic hamsters are used. However, significantly smaller dimensions of their brain structures, if compared to human brain, constrain the implantation possibilities. In case of the entopeduncular nucleus (EPN), the equivalent of the globus pallidus internus in rodents, or the subthalamic nucleus (STN), even the exact targeting leads to a considerable neurological damage inflicted by the electrode lead. Therefore, the stimulating electrode is often placed cranial to the targeted nucleus. However, the resulting proximity of the electrode to the thalamus and the internal capsule (IC) could cause undesirable effects of the stimulation, which must be avoided, as they would distort the experimental results, whose interpretation thus becomes very difficult. The primary goal of this study is to evaluate the theoretical applicability of several electrode designs for the stimulation of the caudally located nuclei and suggest an optimal solution for a precise focalization of the electric field in the target.

## Methods

### Volume Conductor Model

**Figure 1:**
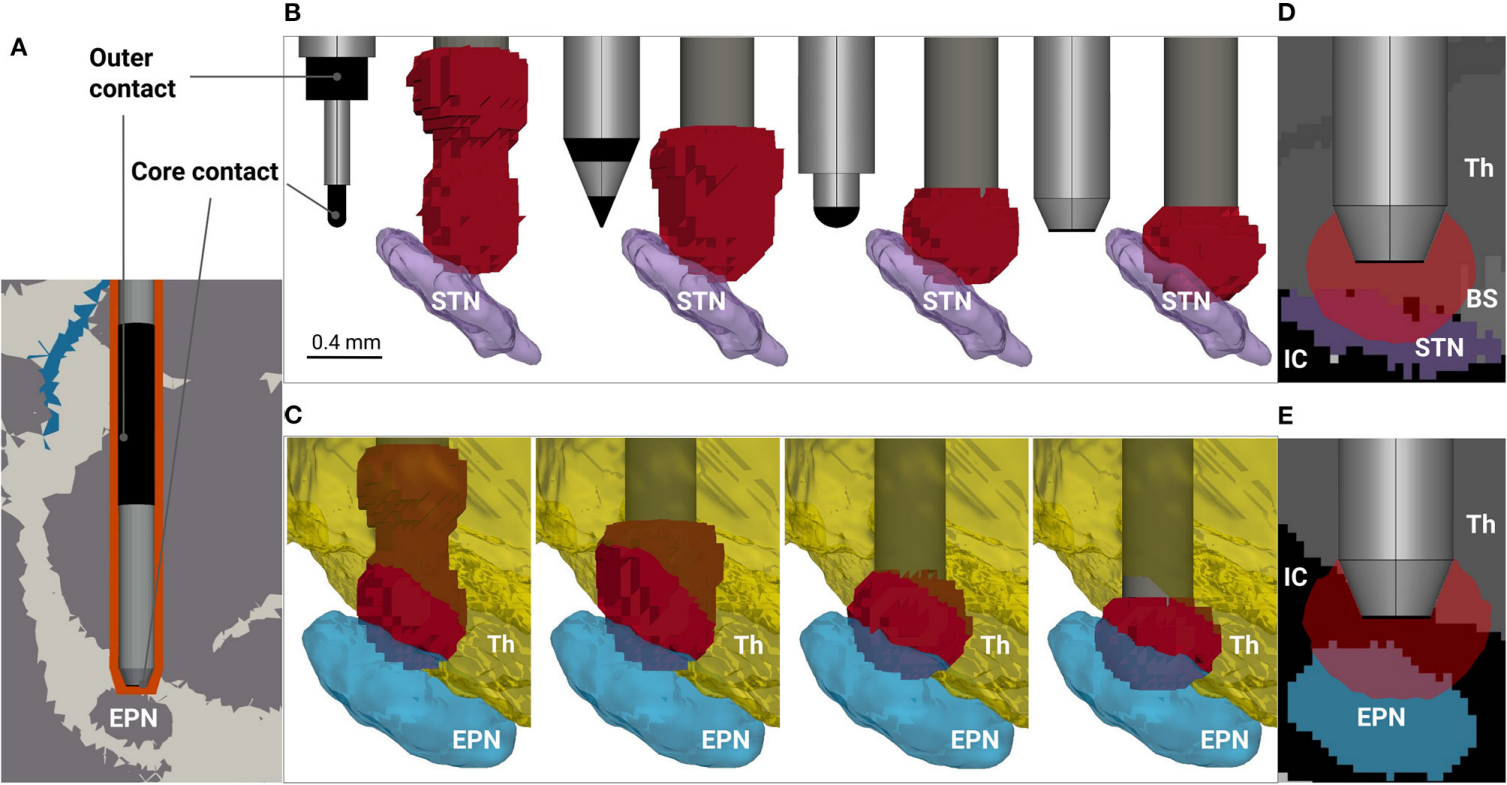
Modeling rodent deep brain stimulation for different electrode designs. **(A)** Fragment of the volume conductor model. The distribution of cerebrospinal fluid, white and gray matter (blue, white and gray colored, respectively), is based on Papp et al. (2014), and the electrode's encapsulation due to inflammation and subsequent scarring is depicted in orange. The electrode tip is placed 0.4 mm above the entopeduncular nucleus (EPN) to avoid neurological damage of the target. The electrode contacts are highlighted in black, while the rest of the surface is insulated. **(B)** Electrode designs (from left to right: SNEX-100, CEAX-100, spherical rounding, blunt end) and predicted neural activation by |***E***| >0.323 V/mm in the vicinity of the subthalamic nucleus (STN), coronal view. For visualization purposes, the thalamus (Th) and the zona incerta are hidden. The roughness of the activation surfaces is due to the resolution of the array points, at which the electric field is probed. **(C)** Predicted neural activation in the vicinity of the EPN. For visualization purposes, the internal capsule (IC) between the EPN and the thalamus is hidden. Directly induced neural activation of the thalamus is predicted for all four electrodes, but the effect is considerably higher for SNEX-100 and CEAX-100. **(D,E)** 2-D sagittal views at the predicted neural activation for the STN and the EPN stimulation, respectively. The former reveals a possible activation in the brain stem (BS), while the latter indicates an activation in the portion of the IC.

For DBS, the electric potential distribution in brain tissue can be approximated by the electro-quasistatic formulation of Maxwell's equations (Plonsey and Heppner, 1967):

(1) $$\begin{array}{l} \nabla \cdot \left[\left(\sigma \left(\text{r},\omega \right)+j\omega \varepsilon \left(\text{r},\omega \right)\right)\nabla \underline{\varphi }\left(\text{r}\right)\right]\;=\;0 \end{array}$$

where $\underline{\varphi }$ is the complex electric potential, σ and ε are the conductivity and the permittivity of the material, ω denotes the angular frequency and “*j*” is the imaginary unit. Since the dielectric properties of brain tissue are frequency dependent (Gabriel et al., 1996), the Fourier Finite Element method (Butson and McIntyre, 2005) is applied, where Equation (1) is solved over the power spectrum of the DBS signal. The amount of computations can be reduced using the octave band method (Butenko et al., 2019). Another issue is the high variation of reported brain conductivities in literature (McCann et al., 2019). In this study, we define the conductivities of gray and white matter as the averages of the values reported by Gabriel et al. (1996) and Koessler et al. (2017) at 50 kHz, and adjust them over the frequency domain according to Gabriel et al. (1996). The heterogeneity of brain tissue, in particular, gray and white matter conductivities, as well as cerebrospinal fluid (2 S/m), is accounted for by mapping the segmented Waxholm space atlas of the Sprague Dawley rat brain (Papp et al., 2014) onto the computational domain (Figure 1A). Furthermore, the anisotropy of the brain tissue is modeled with conductivity tensors derived from diffusion-weighted imaging data (Johnson et al., 2012) and scaled using the normalized mapping approach (Güllmar et al., 2010). Additionally, we include a 0.1 mm encapsulation layer, where a neural degeneration is assumed (Kelly et al., 2017). The dielectric properties of the layer do not significantly affect the current-controlled stimulation (Butenko et al., 2019), and thus it is simply treated as isotropic gray matter.

The Dirichlet boundary conditions for (1) are given by the potentials on the exposed electrode contacts, while the rest of the external surfaces are assumed insulating (∇$\underline{\varphi }$·***n*** **=** 0 with the outer normal vector ***n***). In case of the current-controlled stimulation, the electric potential distribution can be scaled to match the required current using the linearity of (1). In this study, we simulate a 60 μs 60 μA rectangular pulse delivered by a bipolar electrode implanted 0.4 mm above the center of mass of the targeted nucleus.

### Neural Activation

In this study, the neural tissue activation is primarily approximated by the magnitude of the electric field (|***E***| = |∇$\underline{\varphi }$|) and secondly by its divergence (|∇·***E***|) as described in Åström et al. (2015). An alternative to such approximations are mathematical models of neurons, which, however, require not only specification of multiple neural parameters, but also a comprehensive analysis of histological data and fiber tractography. The arising complexity inevitably increases the overall uncertainty of the modeling, and might obscure the electrode performance. Thus, we choose simpler estimators that are directly derived from the electric field. The thresholds above which the tissue is considered activated are 0.323 V/mm and 0.309 V/mm^2^ for |**E**| and |∇·***E***|, respectively. They correspond to the median values over different voltages defined in Åström et al. (2015) for 2.5 μm axon diameters. The median values are chosen due to the high differences of impedances among the considered electrodes. The performance of the electrodes is evaluated by the ratio of the predicted neural activation in the targeted nuclei (the STN and EPN) to the total activated volume. To compute the ratio, we probe the estimators at the points seeded with 0.05 mm resolution in the 2 mm vicinity of the lower electrode (core) contact. All the procedures described above were conducted within the open-source simulation platform OSS-DBS (Butenko et al., 2020); the source code of the project is available at http://doi.org/10.5281/zenodo.4280723.

### Electrode Types

Bipolar electrodes such as SNEX-100 and CEAX-100 from MicroProbe Inc. (MD, USA) are widely used for DBS in rodent models. However, for cranial implantations, where the electrode is not placed in the target, their application may raise the problem that current will bypass the target nucleus to a significant extent. In this particular case, the electric field is shifted upwards which can cause additional direct stimulation effects in the thalamus (Figures 1B,C). This phenomenon can be diminished using monopolar electrodes, but for rodent models certain issues arise due to the application of a ground electrode. For example, intracranial grounding might be strictly limited in size, position and material, which in turn can lead to high electric fields and corrosion on its surface. On the other hand, extracranial grounding can significantly alter the current path due to the low conductive scull tissue and fixation materials, thus adding additional uncertainties to the total impedance. Therefore, we propose a new design for the bipolar electrode based on CEAX-100, but shifting the outer contact to the upper part of the shaft (Figure 1A). The exact position and the length of the outer contact are chosen based on preliminary electrical field simulations and allow to avoid large field amplitudes near its surface. Secondly, different shapes can be considered for the tip of the core electrode. Taking into account its relatively small diameter (0.125–0.250 mm), and, consequently, the processing complexity, we investigate two designs: a spherical rounding and a blunt end (Figure 1B). For the latter, the platinum/iridium core is exposed only at the bottom of the electrode and covered laterally by the insulating tapering that is required to facilitate the implantation. For completeness, we also tested a concave tip, which manufacturing, however, is more challenging. The design did not demonstrate an improved performance in comparison to the blunt end, and thus was excluded from the study. The detailed information on the designs is presented in Supplementary Material.

## Results and Discussion

The analysis reveals a distinct difference in performance among the electrodes (see Table 1, Figures 1B,C). For |***E***| metric, stimulation with SNEX-100 shows the expectedly lowest targeting: the size of the outer contact and its proximity to the core contact create the electric field large enough to expect the neural activation occurring along the whole current path. The same is observed for CEAX-100, but since the contacts are located more closely, the activation in the non-targeted regions is reduced. One possible downside is that due to the sharp tip, this electrode design will create relatively large current flow at this site, which may lead to additional damage due to electrophoresis effects or outright coagulation. The modified design with the spherical rounding shows a distinctly better performance: the remoteness and the large surface of the outer contact diminish the electric field away from the core contact, thus preventing the neural activation in the superior brain regions, in this case the ventral portion of the thalamus (including the zona incerta). However, for these three electrodes, the predicted activation in the target is negligibly small (Figures 1B,C), especially for the STN. This parameter is significantly increased when using the blunt end design (Figures 1B–E). For comparison, the predicted activated volume in the STN is 0.173 and 0.362 mm^3^ for the spherical rounding and the blunt end, respectively. For the EPN, the corresponding values are 0.371 and 0.602 mm^3^. At the same time, the blunt end core contact, insulated on the sides, generates even lower fields above the target. A more conservative but still compelling improvement is estimated by |∇·***E***|. In general, this metric predicts a lower activation: 0.045 and 0.117 mm^3^ in the STN and 0.218 and 0.318 mm^3^ in the EPN for the spherical rounding and the blunt end designs, respectively. This leads to the conclusion that while a relative performance of the electrodes can be assessed with the electric field metrics, accurate predictions of the neural activation require application of detailed neuron models. It can be also noted that for all electrodes and both field metrics the targeting of the EPN is more efficient, which is explained by its smaller lateral extent in comparison to the relatively oblate shape of the STN.

**Table 1:** Electrode performance assessed as a share (%) of the target (STN/EPN) in the total activated volume predicted by the electric field metrics.

**Electrode type**	**|E|**	****|∇·*E*|****
SNEX-100	3.5/9.9	3.0/20.4
CEAX-100	6.0/13.5	2.5/16.7
Spherical rounding	16.4/28.3	23.3/38.3
Blunt end	34.2/48.3	27.5/49.6

As previously mentioned, the DBS electrode not only stimulates neural structures, but also inflicts mechanical damages on brain tissue. For the implantation positions considered in the study, the damage occurs mostly in the cortical and the thalamic areas. Its extent in the ventral portion of the thalamus differs depending on the electrode type, and this factor must be considered by researchers. Among the presented designs, the electrode with the blunt end is expected to inflict the largest damage. This is not necessarily the worst case scenario: in principle, a limited neurological damage may lead to more predictable effects rather than an undesired fragmentary stimulation.

In this study, the diameter of platinum/iridium core for the modified electrodes was set to 0.240 mm. Depending on the target dimensions, the parameter can be adjusted, but it is important to keep in mind that additional neural damage can occur if a certain charge density per phase limit is exceeded (McCreery et al., 1990) or the electrode impedance is too high. The latter will also lead to a quick battery depletion in case of a current-controlled stimulation. The study does not report the computed impedances, since the major low-frequency contributor, the electrical double layer, was not modeled. This parameter and the electrode impedances will be investigated in the upcoming *in vitro* and *in vivo* studies.

In conclusion, we propose two improved designs optimal for focal stimulation of small rodent brain nuclei—a spherical tip likely inflicting less damage, but also resulting in about 50% less focal stimulation, and a relatively blunt one offering the arguably best focal stimulation at the cost of slightly larger tissue damage. In either case, the manufacturing advantage may be seen in the fact that the reference electrode, i.e., the outer contact, does not need any sophisticated design.

## Data Availability Statement

The raw data supporting the conclusions of this article will be made available by the authors, without undue reservation.

## Author Contributions

The study design and the simulations, as well as the preparation of the manuscript were conducted by KB. UR supervised the study, reviewed, and edited the manuscript. RK provided an expert opinion on the biological and anatomical aspects and reviewed the manuscript. All authors contributed to the article and approved the submitted version.

## Conflict of Interest

The authors declare that the research was conducted in the absence of any commercial or financial relationships that could be construed as a potential conflict of interest.
