# Supplementary figures and images for: Numerical Study on Electrode Design for Rodent Deep Brain Stimulation With Implantations Cranial to Targeted Nuclei
Source: Front Comput Neurosci. 2021 Feb 2;15:631188. doi: 10.3389/fncom.2021.631188 (PMC7884621; doi:10.3389/fncom.2021.631188)

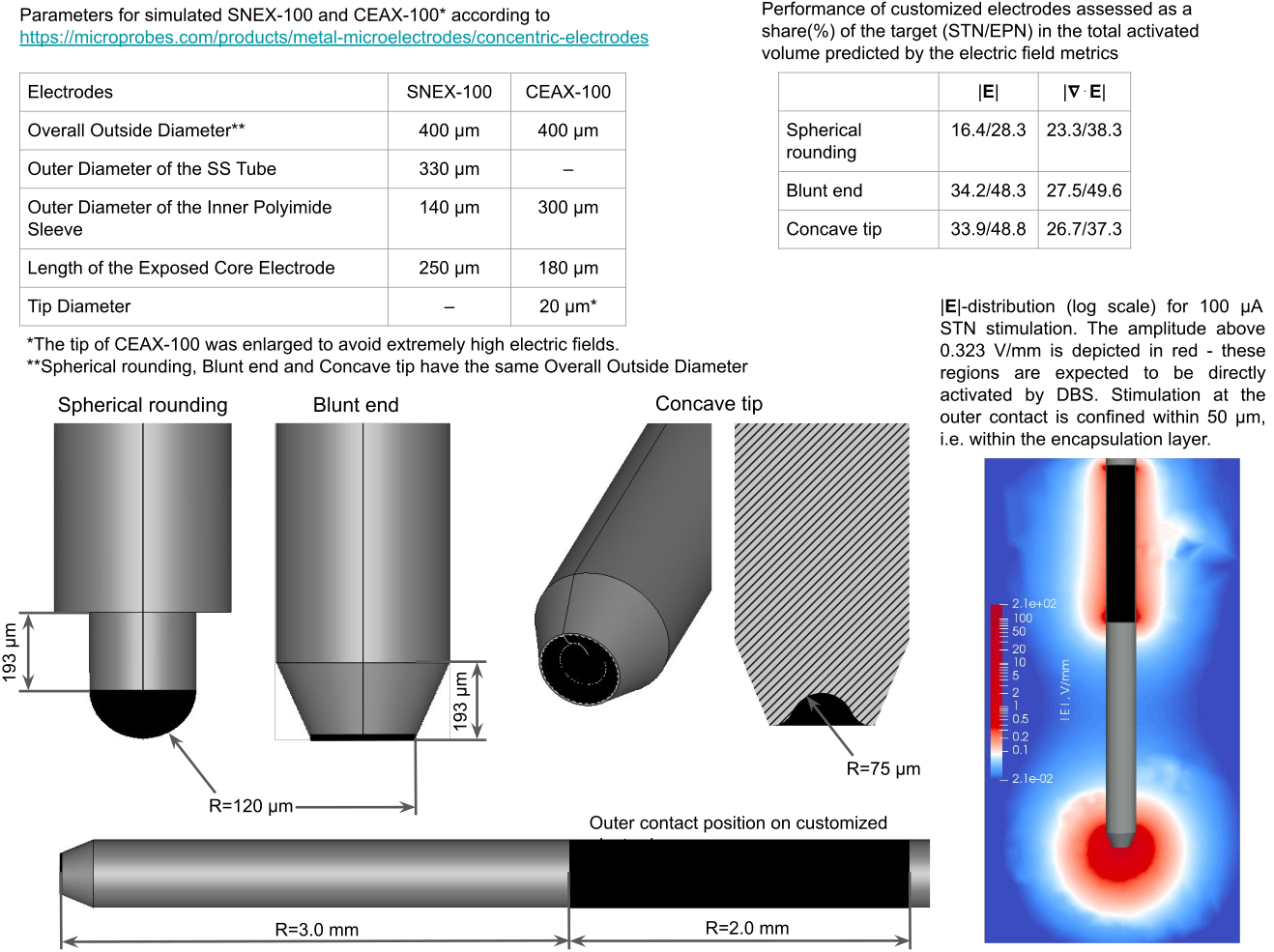

Supplement: Supplementary file 1 [file Image_1.TIF]
